# Solar Light Photocatalytic CO_2_ Reduction: General Considerations and Selected Bench-Mark Photocatalysts

**DOI:** 10.3390/ijms15045246

**Published:** 2014-03-25

**Authors:** Ştefan Neaţu, Juan Antonio Maciá-Agulló, Hermenegildo Garcia

**Affiliations:** Instituto Universitario de Tecnología Química CSIC-UPV, Universidad Politécnica de Valencia, Av. De los Naranjos s/n, 46022 Valencia, Spain; E-Mails: stefan_neatu@yahoo.com (S.N.); j.a.macia.agullo@gmail.com (J.A.M.-A.)

**Keywords:** photocatalytic CO_2_ reduction, solar fuels, heterogeneous photocatalysis, renewable energy

## Abstract

The reduction of carbon dioxide to useful chemicals has received a great deal of attention as an alternative to the depletion of fossil resources without altering the atmospheric CO_2_ balance. As the chemical reduction of CO_2_ is energetically uphill due to its remarkable thermodynamic stability, this process requires a significant transfer of energy. Achievements in the fields of photocatalysis during the last decade sparked increased interest in the possibility of using sunlight to reduce CO_2_. In this review we discuss some general features associated with the photocatalytic reduction of CO_2_ for the production of solar fuels, with considerations to be taken into account of the photocatalyst design, of the limitations arising from the lack of visible light response of titania, of the use of co-catalysts to overcome this shortcoming, together with several strategies that have been applied to enhance the photocatalytic efficiency of CO_2_ reduction. The aim is not to provide an exhaustive review of the area, but to present general aspects to be considered, and then to outline which are currently the most efficient photocatalytic systems.

## 1. Introduction

Due to the shortage in the reserves of oil and natural gas as well as the strong dependence of developed countries on fossil fuels, there is considerable interest in the development of renewable energy resources. One of the primary and inexhaustible energy sources is sunlight reaching the earth’s surface [1]. However, two of the main problems of sunlight, as a source of energy, are the circadian cycle between day and night together with the dependence on seasons and weather conditions and the low sunlight power that makes it necessary to accumulate the energy for long periods with large surfaces before the use of this energy. In this regard, considering the problems associated with the direct use of sunlight, there are two general strategies to use sunlight as a primary energy source, both of them based on the accumulation of solar energy. The first one consists in converting photons from the sun into electrical energy that can be stored in batteries or supercapacitors as well as in any other form of conventional mechanical energy already existing for electricity storage [2]. Particularly for transportation, where high energy powers are required, there is a necessity to accumulate sunlight into an intermediate energy vector that can be, for instance, charge capacitors or batteries [3]. One of the main problems associated with this strategy is the limited resources of lithium and other metals that are currently employed in batteries, making sustainability problematic. An alternative to store sunlight as electrical energy is to convert photons from the sun into chemical energy. The concept of solar fuels refers to the production of chemicals that can release chemical energy using sunlight as a primary energy resource. Among these solar fuels, the one that has attracted the largest attention has been hydrogen produced from water. While the US Department of Energy has identified hydrogen as the ideal renewable fuel for transportation, there is a lack of basic knowledge and technology that still limit the widespread application of hydrogen as fuel [4]. The advantages of hydrogen are its high energy power per mass unit and the lack of negative impact on the environment since water is the only by-product. However, the fact that hydrogen is a gas that cannot be liquefied is associated with problems related to the storage under ambient conditions of reasonable volumes of this gas as well as problems derived from the risk due to flammability and explosion.

Since we are still far from the hydrogen technology era, one possibility, that could represent at least an intermediate situation between the present scenario based on massive consumption of fossil fuels and the future use of hydrogen as energy vector would be solar fuels based on CO_2_ reduction. The aim of the present article is not to provide an exhaustive review of photocatalytic CO_2_ reduction, but to present some general considerations that apply specifically to CO_2_ reduction, particularly in comparison to hydrogen generation from H_2_O, and to describe which are among the most efficient photocatalytic systems reported so far. The reader is referred to several existing reviews for an exhaustive coverage of solar fuel production and, specifically photocatalytic CO_2_ reduction, since herein we focus on reports having promisingly high efficiency. In the next section we comment on the general features associated with photocatalysis related to the reduction of CO_2_ focused on the production of solar fuels.

## 2. Solar Fuels Derived from CO_2_

While water reduction only affords hydrogen, there are a series of products that can be formed from CO_2_ reduction. The list includes oxygenated C_1_ and C_2_ compounds, such as oxalic acid, formic acid, formaldehyde, methanol and CO. In addition to these compounds, methane and C_2_–C_4_ saturated and unsaturated hydrocarbons can also be obtained. From these potential products, the one that would be more valuable as transportation fuel is methanol, considering the high energy content of this alcohol, the convenience of using a liquid compound with a relatively high boiling point and the fact that methanol can even be combined as an additive of fossil fuels used in standard automotive engines. Methanol has a high octane number and can even be the precursor of other gasoline additives for octane number boost, such as methyl *tert*-butyl ether and methyl *tert*-amyl ether. The energy content of oxygenated derivatives decreases as the oxygen percentage in the compound increases. Equations (1)–(3) summarize the combustion free energy of some solar fuels, calculated from the data for the gaseous states from ref. [5]. One point of concern is the so called “carbon footprint” of a process that, in the case of methanol combustion, would be in principle zero if methanol is obtained from CO_2_ reduction by water. The carbon footprint is a quantitative indicator of the influence of a given process on climate change measured by the release of CO_2_ equivalents to the atmosphere. Neglecting the preparation of photocatalysts and other, in principle minor, contributions due to the transportation and manipulation of components, the use of methanol as fuel would not produce any increase of atmospheric CO_2_ if this chemical is obtained from CO_2_. It should be noted that methanol is far more reactive than CO_2_ under photocatalytic conditions, quenching holes and undergoing oxidation to formaldehyde, formic acid and eventually to CO [6–8]. For this reason, it is highly unlikely that in batch reactions the concentration of methanol could be high enough to be valuable, unless special conditions are applied.

(1) $${\text{CH}}_{4}+2{\text{O}}_{2}\to {\text{CO}}_{2}+2{\text{H}}_{2}\text{O}\Delta H^{0}=-802\;\text{kJ}\cdot {\text{mol}}^{-1};\Delta G^{0}=-801\;\text{kJ}\cdot {\text{mol}}^{-1}$$

(2) $${\text{CH}}_{3}\text{OH}+3/2{\text{O}}_{2}\to {\text{CO}}_{2}+2{\text{H}}_{2}\text{O }\Delta H^{0}=-676\;\text{kJ}\cdot {\text{mol}}^{-1};\Delta G^{0}=-690\;\text{kJ}\cdot {\text{mol}}^{-1}$$

(3) $$\text{HCHO}+{\text{O}}_{2}\to {\text{CO}}_{2}+{\text{H}}_{2}\text{O }\Delta H^{0}=-527\;\text{kJ}\cdot {\text{mol}}^{-1};\Delta G^{0}=-520\;\text{kJ}\cdot {\text{mol}}^{-1}$$

Besides methanol, the second most interesting solar fuel in the list of possible products from CO_2_ reduction would be methane. The main advantage of methane is that all the technology for natural gas processing that is presently implemented could be still used independently of the origin of methane, either from natural gas or obtained photocatalytically from CO_2_. In addition, since methane is the most reduced product from CO_2_, its energy content is the highest possible (Equation (1)) and therefore methane is highly attractive due to its energy density. The main drawback, however, of the use of methane is its gaseous state at ambient conditions and the high risk associated with gas use. However, considering the large network of gas pipelines currently available, large scale production of methane could be currently viable.

The above considerations raise a problem associated with solar fuel production from CO_2_, *i.e.*, the need to control product selectivity in achieving a high yield of the target compound. In this regard, it has to be said that, at present, we lack sufficient understanding of the reasons why some processes exhibit different product distributions than others. Below, however, we comment on some trends related to the control of the product distribution that appear to be general, although rationalization is still lacking.

From the point of view of solar fuel production, the ideal process would be the reduction of CO_2_ by water that would correspond to the reverse of Equations (1)–(3). Particularly important, as already mentioned, would be the reverse of Equation (2). However, this process is highly unfavourable from the thermodynamic and kinetic point of view. For this reason and compared to photocatalytic water reduction, photocatalytic CO_2_ reduction is less favourable. From the kinetic point of view, hydrogen generation from H_2_O is mechanistically much simpler than CO_2_ reduction, which, depending on the product formed, always requires several electrons and also probably protons, to form the products. For instance, methanol formation from CO_2_ requires six electrons and six protons that in the reaction mechanism have to be supplied in several consecutive steps thus involving intermediates of several species. In contrast to this, hydrogen generation from water is not only thermodynamically less unfavourable than any of the products resulting from CO_2_ reduction, but also faster. The simplest mechanism for hydrogen generation involves a transfer of one electron from the conduction band of TiO_2_ to a proton leading to a hydrogen atom that will recombine with another hydrogen atom without any activation energy.

The main problem of CO_2_ reduction is that, on one hand water is the most suitable hydrogen donor for CO_2_ reduction, water acting as hole quencher, but on the other hand water can compete, in principle also favourably for electrons in the conduction band. For this reason, it is frequently observed that photocatalytic CO_2_ reduction by water is accompanied by hydrogen generation. Moreover, generally hydrogen is formed in much higher yields than the total amount of products derived from CO_2_. There are specific features for photocatalytic CO_2_ reduction that makes this process notably different from water splitting (Table 1).

The most successful strategy to deal with the problem of concomitant photocatalytic water reduction of hydrogen prevailing over CO_2_ reduction is by making use of a suitable co-catalyst. As a general rule, platinum is a good centre for hydrogen generation from water and, therefore, it is necessary to avoid this metal or at least to make some alloy to minimize hydrogen generation in the photocatalysts containing platinum. On the other hand, copper, either forming independent particles or in combination with platinum, tends to increase CO_2_ reduction disfavouring hydrogen generation.

One important issue that is related to the use of water as reducing agent is the physical state in which photocatalytic reduction is carried out. The majority of the studies have been carried out in liquid media using water as solvent. In this case, the low solubility of CO_2_ in acid or even at neutral pH values (below micromolar concentration) makes the use of basic pH either necessary or convenient. However, although it is clear that basic pH increases CO_2_ solubility in water, this is not necessarily advantageous since, under these conditions, the real species that are present would be carbonates or bicarbonates (Equations (4) and (5)). Those species are more difficult to reduce than CO_2_ itself and, in this way, the advantages of high solubility, meaning high concentration of substrate around the photocatalyst will be lost. In fact, carbonates and bicarbonates, having negative charges, are good hole-quenchers and can donate electrons to the photocatalyst. This is indicated by Equations (6) and (7) and in this way the overall cycle is the oxidation of hydroxide to oxygen rather than the real reduction of CO_2_. For this reason, in order to avoid the problems of CO_2_ solubility in water at neutral or acid pH values, it could be advantageous to work under gas phase conditions by irradiating under a moist atmosphere of CO_2_ in the presence or absence of inert gases. However, these conditions also give rise to problems particularly considering that some products from CO_2_ reduction are not volatile and can strongly adsorb or deposit on the photocatalyst surface and can act as poisons of the photocatalyst. In this regard, experiments performed under gas phase should not be limited to analysis of the composition in the gas phase; it is also necessary to consider the possibility that oxalic acid, formic acid or even methanol and elemental carbon residues formed are deposited on the solid surface.

(4) $${\text{CO}}_{2}+{\text{OH}}^{-}\to {{\text{HCO}}_{3}}^{-}$$

(5) $${{\text{HCO}}_{3}}^{-}+{\text{OH}}^{-}\to {{\text{CO}}_{3}}^{2-}$$

(6) $${{\text{CO}}_{3}}^{2-}+{\text{h}}^{+}\to {{\text{CO}}_{3}}^{•-}$$

(7) $$2{{\text{CO}}_{3}}^{•-}\to 2{\text{CO}}_{2}+{\text{O}}_{2}+2{\text{e}}^{-}$$

Another problem associated with photocatalytic CO_2_ reduction is the much higher reactivity of the reaction products that in addition tend to be deposited or adsorbed on the solid photocatalyst. Again, this situation is totally in contrast to photocatalytic water reduction in which hydrogen, being an insoluble gas, goes out of the aqueous liquid phase and separates from the photocatalyst. For CO_2_ reduction it is just the opposite, since, as commented earlier, the solubility of CO_2_ in water is rather low at acid pH values, while much more reactive products like methanol are highly soluble and become concentrated in the liquid phase. In fact, photocatalytic methanol decomposition to CO_2_ was one of the favourite test reactions in early times for photocatalysis [9]. In this regard, it is very likely that for experiments under batch conditions a stationary concentration of methanol is achieved and, at that moment, the rate of methanol formation from CO_2_ equals the rate of methanol decomposition to CO_2_. To address this issue of methanol instability, a check that can be easily performed and can justify some of the photocatalytic results is to perform independent experiments in which methanol at concentrations above those obtained in the photocatalytic CO_2_ reduction is added deliberately to the CO_2_ and H_2_O mixture used in the photocatalytic CO_2_ reduction. Then, the evolution of the concentration of methanol is followed upon irradiation to determine if its decrease is due to methanol degradation [6,10–12]. It is likely that similar lack of product stability due to photocatalytic degradation could also apply to other products derived from CO_2_ reduction such as: formic acid, formaldehyde and even CO. All these compounds have been reported to decompose photocatalytically and accordingly a stationary maximum concentration should be expected for them in batch experiments [13].

Another point of concern is that in those cases in which the concentration of products in the photocatalytic CO_2_ reduction is low, the products could not have been derived from CO_2_, but from impurities present on the surface of the photocatalyst. TiO_2_ and other metal oxide semiconductors, due to their small particle size and large surface area, can adsorb airborne organic compounds that due to contact with the photocatalyst and their higher reactivity with respect to CO_2_ could be the origin of the photoproducts, leading to mistakes about the activity of the photocatalyst for CO_2_ reduction [14–16]. In these cases, in which the weight of photocatalyst in the experiment is high and the product concentration is low, it is advisable to submit the photocatalyst to calcinations to remove the organic material that could be present on its surface, prior to the photocatalytic experiment.

## 3. Photocatalyst Design for CO_2_ Reduction

In order to be efficient, one photocatalyst must encompass several features, some of them common for any catalyst, such as high surface area and Brönsted centres for fast proton transfer. In addition, a photocatalyst should combine the properties of the semiconductor with other new desirable properties (Table 2).

**Scheme 1.**
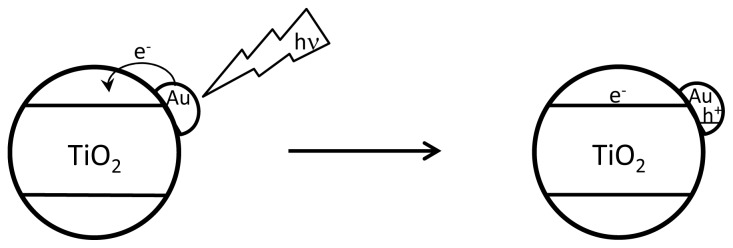
Proposed mechanism for photoexcitation of TiO_2_ by irradiation of Au nanoparticles (NPs).

These desired properties are related to light absorption, modification of the activity of the semiconductor and the presence of additional components acting as co-catalysts. With respect to light harvesting, the optimal photocatalyst should absorb efficiently over the whole wavelength range of the solar spectrum. However, this property has not yet been achieved and, in fact, TiO_2_ and other common semiconductors only absorb directly a negligible amount of solar energy. For this reason, light harvesting centres having the role of absorbing solar photons and transferring electrons to the semiconductor are needed. Among the possible light harvesters, organic molecules are highly efficient, but tend to decompose and for this reason organic dyes are in general considered not suitable due to low stability even though their initial activity can be very high. Transition metal complexes exhibit in general higher stability, but also the presence of organic ligands raises concerns about long term stability. Metal polypyridyls such as ruthenium tris(bipyridyl), porphyrins and metal phthalocyanines are among the preferred metal complexes for solar light harvesting [17]. In this context, much more preferable is the use of a totally inorganic light harvesting centre and noble metal nanoparticles (NPs) having a surface plasmon band in the visible range as well as high photocatalytic stability, have demonstrated high capability and durability. Some metal NPs, such as gold, silver and copper, when of appropriate size and morphology, exhibit absorption bands in the visible region as a consequence of the collective oscillation of electrons on the surface of the NPs (“surface plasmon band”). In particular, the surface plasmon band absorption of Au NPs appears as a very broad band at λ max around 560 nm expanding from 400 to 700 nm [18,19]. The exact position of the λ max of the band depends among other factors on the dielectric constant of the support, the average particle size of Au NPs, the charge density, either positive or negative, on the Au NP and some other parameters [20,21]. Specifically, Au NPs supported on titania, having characteristic pink-purple colour due to the presence of Au, has considerable importance as a thermal catalyst for different types of reactions, including low temperature CO oxidation, selective oxidation of alcohols, selective nitro group reduction and epoxide rearrangement, among others [22–25]. Due to this interest in catalysis, several reliable methods for preparation of Au/TiO_2_ have been reported and, in fact, this material has become a reference catalyst available from the World Gold Council to ensure the consistency and reproducibility of catalytic results between different laboratories. This commercially available reference catalyst is prepared by depositing on Degussa P25 TiO_2_ Au NPs between 4–7 nm using the so-called “deposition-precipitation method” initially developed by Haruta [22]. In the deposition-precipitation method a solution of AuCl_4_^−^ is contacted at pH > 5 with TiO_2_ over a long time allowing anchoring of the AuCl_4_^−^ species on the surface of the TiO_2_. Subsequently, the solid is recovered and reduced thermally or chemically by using alcohols or even hydrogen gas. This Au/TiO_2_ has been found to be an excellent photocatalyst for the solar light generation of hydrogen from a water-methanol mixture [26]. It is proposed that this visible light photocatalytic activity derives from Au NPs acting as light harvester centres that upon absorption of one photon eject hot electrons that are able to populate the conduction band of TiO_2_ (Scheme 1). Similarly, these types of materials containing noble metal NPs or their alloys can act as photocatalysts for CO_2_ reduction as is mentioned later in this paper for the combination of platinum and copper(I) oxide.

A different approach to introduce visible light photo-response in TiO_2_ and other wide band gap semiconductors is doping by metals or non-metallic elements. While TiO_2_, being a simple metal oxide, has excellent photoactivity upon UV light irradiation, the aim is to expand its photoactivity as much as possible towards longer wavelengths without causing detriment to its activity. In the doping process, the aim is to reduce the band gap of the semiconductor by introducing extra levels in the gap region. These levels can correspond to empty orbitals below the conduction band energy of the semiconductor (case of metal doping) or by introducing additional occupied orbitals above the valence band of the semiconductor. The main problem of doping is reproducibility of the photocatalytic activity, since it has been found that there is an optimal doping level and higher concentrations of the dopant element can be extremely detrimental since, besides acting as light harvesters, the doping elements can also act as recombination centres [27,28]. Charge recombination is the main deactivation pathway competing with photocatalytic activity and should be avoided or minimized as much as possible. What happens is that most of the doping procedures, *i.e.*, sol-gel or doping in the solid state, require some calcination step and in this treatment the dopant element can be grafted to or expelled from the framework of the semiconductor, making it extremely difficult to know in advance what is going to be the exact amount of the dopant that will remain in the material. In addition, all the doped semiconductors must be checked for long-term photocatalytic stability since very frequently the activity of the fresh material decays during operation due to the spontaneous migration and relocation of the dopant element during the photocatalytic reaction. It should be taken into account that photocatalysts are subject to considerable framework stress as a consequence of the continued change in the oxidation state of the elements involved in the electron/hole separation/recombination.

Besides light harvesting centres, co-catalysts, meaning some additive or modifier deposited on the semiconductor surface, are known to play a crucial role in the photocatalytic efficiency. Once charge separation and migration of the charge carriers has taking place, electrons and holes are considered to reside preferentially in different centres generally denoted as “traps”. The photocatalytic event requires that these trapping sites are present on the external surface of the particle exposed to substrates and reagents. Once on the external surface, they should be transferred to the substrates at high reaction rates. It should be mentioned that charge accumulation or charge unbalance in a semiconductor particle always stops or disfavours charge separation with respect to a neutral particle. Thus, the Fermi levels of the semiconductor will depend on the state of charge and this means that an adequate management of charge transfer, either electrons or holes, from the particle to the substrate can determine the rate of the other events in the photocatalytic mechanism.

## 4. Heterogeneous Photocatalysts for CO_2_ Reduction

There are two main types of heterogeneous photocatalysts currently employed in the CO_2_ reduction process: bulk semiconductor photocatalysts and so-called matrix-dispersed photocatalysts [29–33]. Among all the bulk semiconductor photocatalysts engaged in the CO_2_ reduction, which comprise metal oxides, sulphides, nitrides and oxynitrides, titanium dioxide is the most used material; its photocatalytic activity being enhanced especially when appropriate co-catalysts, usually noble metals, are employed. In the second category of matrix-dispersed photocatalysts, the photoactive material is isolated or entrapped in an inert matrix, which provides extremely good adsorption capacity and large surface area. Both categories are briefly discussed below.

In the case of matrix-dispersed photocatalysts, one photocatalytic system that has been shown to be among the highest activity for photoreduction of CO_2_ to methanol has been the one in which TiO_2_ NPs are incorporated inside the mesopores of SBA-15 silica [34]. SBA-15 ranges among the periodic mesoporous silicas with the highest porosity and largest surface area that typically goes above 1000 m^2^/g. In addition, one advantage of SBA-15 is its high stability in the presence of water compared to other mesoporous silica and MCM-41 in particular, which is notoriously unstable when suspended in water. The large pore volume, surface area and adsorption capacity characteristic is the reason why SBA-15 has been frequently used to prepare composite materials in which a photoactive guest is incorporated inside the rigid matrix provided by SBA-15. In the present case, TiO_2_ NPs have been entrapped inside the mesopores of the SBA-15 (around 10 nm size) by performing an *in situ* hydrolysis and condensation of titanium tetra-isopropoxide in isopropanol, obtaining samples containing between 25–65 wt % TiO_2_. The crystallinity of TiO_2_ NPs was increased by calcination of the samples at temperatures between 500–550 °C.

**Scheme 2.**
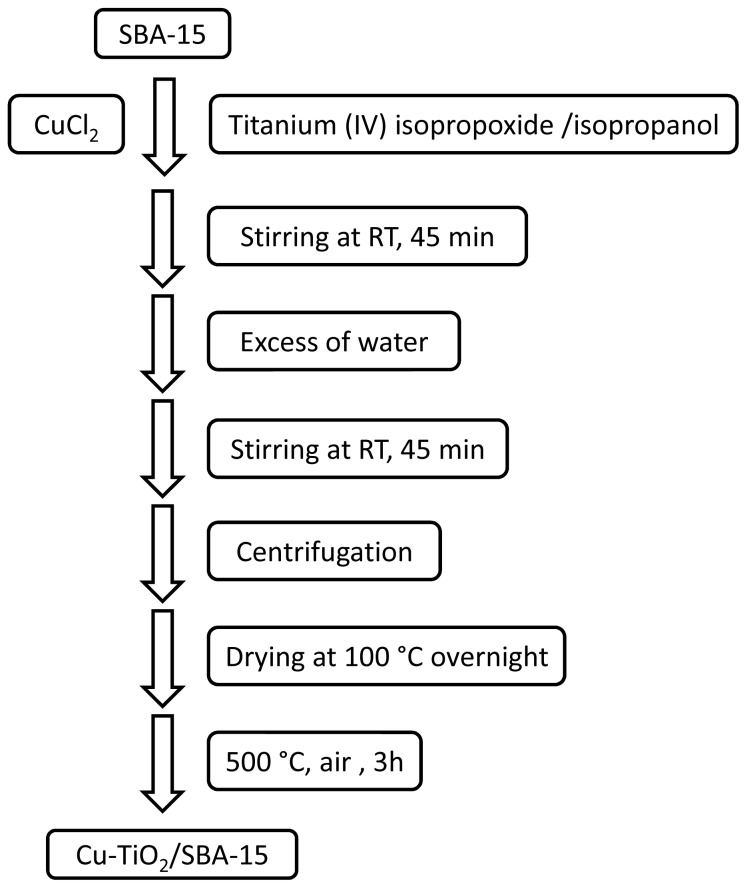
Steps in the preparation of copper modified TiO_2_ NPs included in the channels of SBA-15.

The preparation procedure can be adapted to obtain samples in which copper is also present as co-catalyst by simply adding CuCl_2_ (2 wt %) to the isopropanol solution in which the sol-gel formation of the TiO_2_ NPs is taking place. Scheme 2 summarizes the preparation route leading to Cu-TiO_2_/SBA-15. Characterization of the resulting Cu-TiO_2_/SBA-15 shows that the crystallinity of the SBA-15 has largely remained after the preparation of TiO_2_ which is mainly in the anatase phase. Particularly notable is the high crystallinity of the anatase in which copper is present. The resulting Cu-TiO_2_@SBA-15 photocatalysts were tested for the reduction of CO_2_ in liquid aqueous phase at initial pH = 13. The actual pH of the photoirradiation was around 7, meaning that the prevalent species submitted to photocatalysis were bicarbonates. Comparing with analogous samples in which copper was deposited on TiO_2_, the photocatalytic activity of Cu-TiO_2_/SBA-15 was higher in terms of both initial reaction rate and final productivity of methanol. Table 3 presents the approximated values of methanol production rates for some of the samples that have been tested. The above experiments show how it is possible to increase the photocatalytic activity intrinsic of a semiconductor by adequate structuring and particle size reduction and by embedding the active photocatalytic components in a porous matrix. It should be however noted that these experiments in which the average methanol production rate was 627 μmol·g^−1^·h^−1^ were performed using UV light from a medium pressure metal halide lamp having a maximum light intensity at 365 nm. Thus, modification of this type of photocatalytic system to be active under visible light and solar light irradiation remains to be achieved. These results are certainly remarkable since, as indicated earlier, formation of high concentrations of methanol should not be expected in conventional TiO_2_ photocatalysts due to the fast degradation of this product by oxidation. It seems that in the present case, confinement of the photocatalytic centres in a matrix alters the intrinsic reactivity, probably due to preferential adsorption of CO_2_ *vs.* CH_3_OH near the photocatalytic centres.

**Scheme 3.**
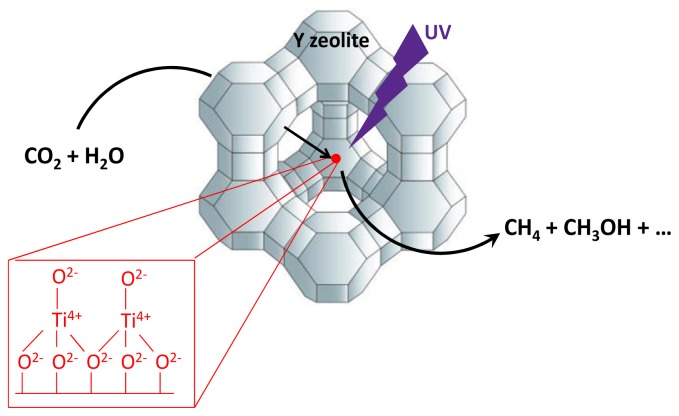
Pictorial illustration of matrix isolated titania clusters encapsulated inside the cavities of zeolite Y acting as photocatalyst for CO_2_ reduction by H_2_O.

Zeolites are microporous solids with a negligible photocatalytic activity, but the introduction of TiO_2_ clusters in their pores or grafted to their framework produces a considerable enhancement in activity. Matrix dispersed TiO_2_ included within the zeolite framework has been prepared using hydrothermal synthesis. Several periodic titanosilicates such as TS-1, Ti-MCM-41 and Ti-MCM-48 have been prepared. TS-1 has a small pore size (*ca.* 5.7 Å) and a bi-dimensional channel structure, Ti-MCM-41 has a larger pore size (>20 Å) but a one-dimensional channel structure and Ti-MCM-48 has both a large pore size (>20 Å) and three-dimensional channels [35,36]. In addition, TiO*_x_*(OH)*_y_* clusters entrapped within the micropores of Y-zeolite have been prepared by ion exchange and impregnation [35,37] (Scheme 3). UV irradiation of the catalysts was carried out using a 75 W high-pressure Hg lamp (λ > 280 nm) at 328 K. UV-irradiation of the photocatalysts in the presence of a mixture of CO_2_ and H_2_O led to the evolution of CH_4_ and CH_3_OH at 328 K, as well as trace amounts of CO, C_2_H_4_ and C_2_H_6_. Among the series of materials prepared Ti-MCM-48 exhibited the highest photocatalytic activity. The higher reactivity and selectivity in the reduction of CO_2_ with H_2_O to produce methanol in the case of Ti-MCM-48 may be the combined contribution of the high dispersion state of the Ti-oxide species and the large pore size having a three-dimensional channel structure.

The effect of Pt-loading on the activity of Ti-MCM-48 and Ti-oxide/Y-zeolite has also been investigated. Although the addition of Pt is effective in increasing the activity of both samples, only the formation of CH_4_ is promoted, accompanied by a decrease in the CH_3_OH yields. The highest value of methane production reported by Anpo *et al.* is 12.5 μmol· (g^−1^ TiO_2_)·h^−1^ corresponding to two samples; namely Pt (1 wt %)-Ti-MCM48 with a ratio Si/Ti = 80 and Pt (1 wt %) and ion exchanged Ti-oxide/ Y-zeolite with 1 wt % as TiO_2_. The highest production of methanol was around 5 μmol·(g^−1^ TiO_2_)·h^−1^ for the sample Ti-oxide/Y zeolite with 1 wt % as TiO_2_ and without Pt.

Among many other matrices used as support to incorporate Ti-oxide, a very recent study used montmorillonite (MMT) to increase the photoactivity of titania [38]. In this study montmorillonite modified titania nanocomposites synthesized by a single step sol-gel method were introduced in a stainless steel reactor filled with a gas mixture of helium, CO_2_ (20%) and water vapour and equipped with quartz windows. The reactor was irradiated with a 500 W mercury lamp. The effect of temperature on the photocatalytic CO_2_ reduction revealed that the highest yield rate of methane (441.5 μmol·g^−1^·h^−1^) and carbon monoxide (103 μmol·g^−1^·h^−1^) is reached at 393 K when 20 wt % MMT/TiO_2_ nanocomposite was used.

A similar influence of the temperature increase caused by irradiation was reported by Grätzel and co-workers in 1987 when they studied the selective production of CH_4_ (selectivity bigger than 99%) from a mixture of H_2_ and CO_2_ in argon, at atmospheric pressure, using highly dispersed Ru/RuO*_x_* loaded onto TiO_2_ as a catalyst [39]. The authors suggested that the whole reaction mechanism comprises the following steps: (i) carbon dioxide reduction by four electrons provided by the titania conduction band taking place on the Ru species and leading to the production of Ru-C species and oxide ions (O^2−^); (ii) hydrogen is oxidized and then reacts with the oxygen ions to form water; (iii) methane is generated during the reaction of hydrogen with the carbidic surface carbon (Ru–C) followed by regeneration of Ru. The methanation of carbon dioxide takes place even under dark conditions but the irradiation of the surface with simulated sunlight boosts the activity of the catalyst leading to a methane production rate of about 116 μL·h^−1^ per 100 g of material.

However, after a comprehensive study of Grätzel’s work, it has been suggested that the photocatalytic reduction of CO_2_ with hydrogen over Ru/RuO*_x_* sensitized TiO_2_ catalyst occurs due to a thermal effect rather than involving a genuine photocatalytic reaction [40]. Actually, since the reaction between CO_2_ and H_2_, indicated in Equation (8), is a downhill reaction (Δ*G*^0^ < 0), in principle, a large range of catalysts could be suitable to promote this process even at room temperature and in the absence of light. The problem arises when considering the solar-driven CO_2_ photo reduction by water, since this process can be envisioned as a combination of photocatalytic splitting of water to provide hydrogen (Equation (9)), followed by thermal methanation (Equation (8)). According to this, one possibility for the overall process of photo-methanation of CO_2_ could be a photocatalytic hydrogen generation and a dark, catalytic methanation of CO_2_.

(8) $${\text{CO}}_{2}+4{\text{H}}_{2}\to {\text{CH}}_{4}+2{\text{H}}_{2}\text{O }\Delta G^{0}=-114\;\text{kJ}\cdot {\text{mol}}^{-1}$$

(9) $${\text{H}}_{2}\text{O}\to {\text{H}}_{2}+1/2{\text{O}}_{2}\;\Delta G^{0}=237\;\text{kJ}\cdot {\text{mol}}^{-1}$$

**Scheme 4.**
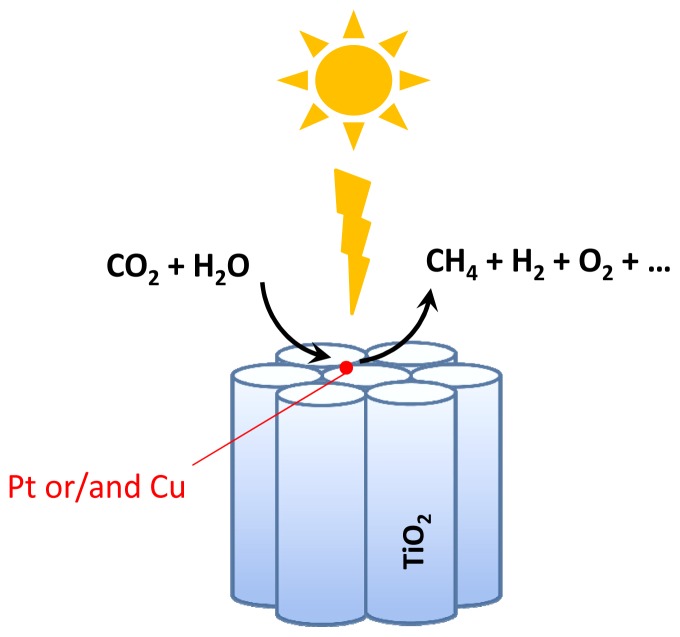
Architecture of N-doped TiO_2_ NT array acting as solar light photocatalyst for CO_2_ reduction by water.

Conventional TiO_2_ materials are constituted by individual NPs in an unstructured way forming a powdered material. Ordering and structuring is one of the general methodologies that can lead to an increase in photocatalytic efficiency. The reason for this is because migration of charge carriers can take place for much longer distances when the material has a preferential morphology like rods or nanotubes. In this context, one of this type of photocatalyst that has exhibited among the highest efficiencies is composed of TiO_2_ nanotube (NT) arrays of about 130 μm lengths, 95 nm pore size and 20 nm wall thickness [41]. These titania NT arrays were doped with nitrogen and in this way the N-doped TiO_2_ NT array absorbs up to wavelengths shorter than 500 nm. Doping with nitrogen takes place spontaneously during the anodization of titanium metal foil using ammonium fluoride as electrolyte under ambient atmospheric conditions. In addition to doping and structuring, the photocatalyst in the form of a porous film was further modified by depositing platinum and copper as co-catalysts. This system of structured array of N-doped TiO_2_ NT with Pt and Cu co-catalyst combines some of the components that are known to increase the photocatalytic activity of semiconductors, including control of the morphology of the semiconductor to favour preferential charge migration, doping to increase visible light photoactivity and the presence of co-catalysts to increase the dark elementary steps related to charge transfer management to the substrates with adequate balance of electrons and holes. The systems studied include a series of TiO_2_ NT arrays containing platinum, copper or platinum and copper in different regions (Scheme 4). These metals were introduced by direct current sputtering. Electron microscopy characterization of the NT arrays shows that in the case of platinum, this metal forms islands of about 40 nm located in the pore mouth of the NTs but without blocking the entrance of the pores. It can be assumed that similar features could be also observed in the deposition of copper, but further characterization including location and size of copper, its distribution in the different oxidation states would be necessary. Notable influence of the co-catalyst on the photocatalytic activity was observed depending on the nature of the metal. In particular, platinum exhibits more than three times higher activity for hydrogen generation while copper has higher efficiency than platinum for hydrocarbon generation (see Table 4). Furthermore, if the UV light from the sun is filtered, no hydrogen is produced using the photocatalyst consisting of Cu deposited on N-doped TiO_2_ nanotube arrays and only CO_2_ reduction is observed. These experimental results, as well as others in the same direction reported in the literature, point to the fact that the presence of Cu NPs on the surface of the photocatalyst enhances the selectivity towards methane *vs.* hydrogen generation or other CO_2_ reduction products.

Other experiments have shown the effect of temperature on the product formation rate. Since increased irradiation intensity is always accompanied by an increase in temperature, the investigation of the nitrogen-doped titania NT array films loaded with platinum and copper under different solar irradiation conditions revealed a significant increase of the total hydrocarbon formation rate from around 90 ppm·cm^−2^·h^−1^ at 1 Sun to about 450 ppm·cm^−2^·h^−1^ under 3.5 Sun, which corresponds to a 5-fold increase in the hydrocarbon yield [42].

Further refinements in this TiO_2_ NT array system, by coating the undoped titania double-walled NT arrays with coaxial Cu-Pt bimetallic layers as well as using a much higher pressure inside the photoreactor, allow improved hydrocarbon productivity of the photocatalyst. Under these conditions the photocatalysts demonstrate at least a fourfold improvement in CO_2_ conversion rates obtaining, under 1 Sun irradiation and room temperature conditions, a hydrocarbon production rate of 574 nmol·cm^−2^·h^−1^ that is among the highest CO_2_ conversion rates reported so far [43]. Even though the CO_2_ conversion rate under these conditions is one of the highest values so far reported, the novelty of this study is also in the effectiveness of these photocatalysts for the reduction of non concentrated carbon dioxide (0.998% in N_2_), these materials being capable of reaching an average hydrocarbon production rate of 610 nmol·cm^−2^·h^−1^.

Such results clearly show that in order to obtain high CO_2_ reduction rates it is essential to design and develop bifunctional co-catalysts with proper nanostructuring and adequate properties. In this respect, a recent work presented the design and preparation of an efficient binary co-catalyst for CO_2_ reduction in the presence of H_2_O [44]. In this work, the preparation of TiO_2_-loaded Pt and Cu binary co-catalysts was performed using a photodeposition technique in which, first the Pt NPs were introduced onto TiO_2_ providing Pt/TiO_2_ with a 0.9 wt % Pt content, and then the deposition of Cu was carried out at different irradiation times leading to the synthesis of several Cu/Pt/TiO_2_ materials. Using this type of preparation procedure, core-shell structures, in which the Pt NPs are covered by a Cu shell, have been developed, the Cu contents varying from 0.59–1.7 wt % after 1 and 5 h of irradiation, respectively. The characterization of the samples revealed that copper was preferentially deposited on Pt NPs and its oxidation state in the shell is one, as Cu_2_O, while the mean size of the core-shell structured particles was around 7.3 nm. The performance of the photocatalytic CO_2_ reduction with water leads to the formation of CO, CH_4_ and H_2_, with the 5 h photodeposited Cu/Pt/TiO_2_ material having the highest selectivity for CO_2_ reduction of 85%.

Concerning the actual reported productivity values it should be commented that these numbers have only relative importance since they depend on different experimental factors and reaction conditions. Thus, the same material can exhibit higher or lower CO_2_ conversion rate depending on factors such as photoreactor design, CO_2_ pressure and concentration, intensity and type of light, photocatalyst concentration and placement inside the photoreactor together with others. Therefore, in order to make a good comparison about the performance of different photocatalysts, future studies in the field of photocatalytic CO_2_ reduction should take into account the ten IUPAC recommendations [45]. For instance, the absorbed radiation should be properly determined (either by actinometry or by proper calculation) and the radiation source should have reached steady-state regime, among others.

## 5. Conclusions and Future Prospects

In this paper we have tried to convey the interest of developing CO_2_ reduction by photocatalytic means in the context of searching for alternatives to the depletion of fossil resources. It is clear that the current state of the art is still far from having identified an optimal photocatalyst for CO_2_ reduction which may be applied commercially. Since solar fuel production derived from CO_2_ is the principal goal, it is obvious that further work in photocatalyst design must combine in an appropriate manner every possibility to improve the solar light response of the photocatalyst. In this context, photocatalytic CO_2_ reduction is highly dependent on photocatalyst modification with co-catalysts to control the efficiency and selectivity of the process.

Application of theoretical calculations and *in situ* spectroscopic techniques will help to improve understanding of the elementary steps occurring in the photocatalytic carbon dioxide reduction process. The *in situ* monitoring of the surface reaction intermediates, the investigation of the rate limiting step and the process dynamics of adsorption and desorption of these species by using temperature-programed studies will boost our knowledge of photocatalytic CO_2_ reduction and finally should lead to the synthesis of new photocatalysts with higher efficiency, selectivity, long-term stability and economic competitiveness. Considering current performance, it is necessary to increase conversion efficiency by several orders of magnitude since the actual conversions are quite low and still far from practical application. In this context, engineering of the photocatalyst including nanostructuring is crucial, offering a good possibility to enhance the overall photocatalytic activity of these materials.

## Figures and Tables

**Table 1.** Some differences between photocatalytic H_2_O and CO_2_ reductions.

Photocatalytic H_2_O reduction	Photocatalytic CO_2_ reduction	How to drive phtotocatalysis towards CO_2_ reduction
H_2_ generation from water	Low CO_2_ solubility in water	Gas phase reaction
Single product	Many possible products	Presence of co-catalysts
Simple mechanism	Mechanism involving several e^−^ and H^+^ transfers	Presence of acid sites
H_2_ diffusing out of the liquid phase	Products in contact with the photocatalyst with decomposition	Continuous flow
Thermodynamically uphill	Thermodynamically much less favorable than H_2_ production	e^−^ with appropriate reduction potential

**Table 2.** Desirable properties of a photocatalyst.

How to accomplish the property	Property	Effect
Small particle size	High surface area	High adsorption
Crystalline material	Single site structure	Homogeneity
Engineering band gap	Light absorption	Higher efficiency
Preferential migration along certain direction	Efficient charge separation	Low recombination
Presence of co-catalysts	Long lifetime of charge separation	Possibility of chemical reactions
High crystallinity	High mobility of charge carriers	More efficient charge separation
Adequate co-catalysts	Selectivity towards a single product	Efficient chemical process

**Table 3.** Photocatalytic methanol formation rate of various photocatalysts at different irradiation times. The values indicated have been obtained by interpolating data from reference [34].

Photocatalyst	Methanol formation rate (μmol·g^−1^) at different irradiation times			

	60 min	120 min	240 min	480 min
TiO_2_	450	900	950	1100
2 wt % Cu/TiO_2_	1500	2500	3000	3200
(2 wt % Cu/TiO_2_)/SBA-15	1600	2850	3010	4100

**Table 4.** Photocatalytic conversion of CO_2_ over nitrogen-doped TiO_2_ NT array films loaded with single or binary platinum and copper co-catalysts under different irradiation conditions. The formation rate values have been estimated by interpolating data from reference [41].

Photocatalyst	Irradiation conditions	Formation rate (ppm·cm^−2^·h^−1^)		

		Hydrocarbon	Hydrogen	Carbon monoxide
Pt/N-TiO_2_ NT	Solar light	85	190	2
Cu/N-TiO_2_ NT	Solar light	105	60	10
	Visible light	30	–	19
PtCu/N-TiO_2_ NT	Solar light	111	160	–
